# An End-to-End Oil-Spill Monitoring Method for Multisensory Satellite Images Based on Deep Semantic Segmentation

**DOI:** 10.3390/s20030725

**Published:** 2020-01-28

**Authors:** Yantong Chen, Yuyang Li, Junsheng Wang

**Affiliations:** Department of Information Science and Technology, Dalian Maritime University, Dalian 116026, China; chenyantong@dlmu.edu.cn (Y.C.); lyy@dlmu.edu.cn (Y.L.)

**Keywords:** sea oil spill, convolutional neural network, semantic segmentation, conditional random field, remote-sensing image

## Abstract

In remote-sensing images, a detected oil-spill area is usually affected by spot noise and uneven intensity, which leads to poor segmentation of the oil-spill area. This paper introduced a deep semantic segmentation method that combined a deep-convolution neural network with the fully connected conditional random field to form an end-to-end connection. On the basis of Resnet, it first roughly segmented a multisource remote-sensing image as input by the deep convolutional neural network. Then, we used the Gaussian pairwise method and mean-field approximation. The conditional random field was established as the output of the recurrent neural network. The oil-spill area on the sea surface was monitored by the multisource remote-sensing image and was estimated by optical image. We experimentally compared the proposed method with other models on the dataset established by the multisensory satellite image. Results showed that the method improved classification accuracy and captured fine details of the oil-spill area. The mean intersection over the union was 82.1%, and the monitoring effect was obviously improved.

## 1. Introduction

In recent years, with the increasing global demand for crude oil, the offshore-oil-transportation industry has rapidly developed. However, there are frequent oil-spill accidents on the sea surface. For example, in 2010, a Gulf of Mexico rig exploded, which led to the largest marine oil spill in history. The accident caused a large area of marine pollution. After an oil-spill accident, it is very important to accurately collect oil-spill information. Therefore, the development of oil-spill monitoring is significant for marine-environment protection.

The traditional method of oil-spill monitoring is aerial or field investigation, but this needs a large workforce and many material resources, which leads to high costs and difficult operations. Remote-sensing satellites have a wide coverage range and can monitor an oil spill throughout days and nights, making it the best means of sea-surface monitoring. At present, the synthetic aperture radar (SAR) is usually used in oil-spill monitoring on the basis of remote-sensing satellites. SAR advantages are wide coverage and all-weather operation, so it can effectively monitor the position of an oil spill. Research on oil-spill monitoring in SAR images has mainly focused on the detection of an oil spill. When the marine environment is complex, such as with a wind-shadow area, upwelling zones, and sea clutter ship wake, this affects the oil-spill area. There are dark areas in a SAR oil-spill image, and this method spends less effort on the calculation of an oil-pollution area. An optical image usually has high resolution and rich colors. Optical remote-sensing oil-spill images can effectively eliminate the interference of the dark areas, and the oil-spill area can be effectively estimated, which is crucial for marine-ecological-damage assessment and oil-spill control.

For oil-spill monitoring, image segmentation is mainly used. The traditional methods of oil-spill segmentation are as follows. (1) The threshold segmentation method [1], which divides image pixels into several classes. This method is simple and requires little computation, but it is easily affected by sea noise and uneven image gray distribution. This results in low segmentation accuracy. (2) Edge-information detection [2], which combines the shape characteristics of an oil-spill area and edge information. Then, the candidate oil-spill area can be obtained. (3) Semantic segmentation [3,4], which clusters pixels belonging to the same category in an image into one region. The oil-spill area and the sea surface can be clearly classified, and semantic segmentation has a more detailed understanding of an image. The traditional classification methods used for semantic segmentation are (1) Markov random fields [5], which is an undirected graph model that defines the mark for each pixel. (2) Random decision forests [6], a classification method that uses multiple trees to train and predict samples. (3) Condition random field [7,8], which represents a Markov random field with a set of input random variables *X* and a set of output random variables *Y*. Among them, the fully connected conditional random field (CRF) overcomes the shortcomings of a traditional CRF that could miss fine structures. However, the classification effect of these traditional methods is still poor.

In recent years, deep learning has been widely used in the field of computer vision. It has achieved breakthrough success, especially in image classification. Deeplab [9,10] was proposed by the Google team for semantic segmentation; the network architecture is Resnet. It uses an atrous convolution to adjust the resolution, which can expand the receptive field and reduce the calculation amount. It also extracts features by deep convolutional neural networks (DCNN) [11,12]. However, there are still some problems with Deeplab. For instance, first, it uses DCNN for rough segmentation. Then, the fully connected conditional random field is used for fine segmentation. The end-to-end connection cannot be realized, which leads to low classification accuracy. Second, the fine-detail extraction of an oil-spill area is poor and time-consuming.

Aiming at the above problems and the characteristics of SAR and optical remote-sensing images, we proposed a new semantic-segmentation model based on Deeplab. It is a multisource remote-sensing-image sea-oil-spill semantic-segmentation model for monitoring oil-spill areas. Combining fully connected CRF with deep convolution neural network, it uses Gaussian pairwise potential and the mean-field-approximation theorem. The CRF was established as a recurrent neural network (RNN) [13], and it is seen as part of the neural network. These could obtain a deep end-to-end network with both DCNN and CRF. The model could be used to monitor the oil-spill area of SAR and optical sensing images; the oil-spill area was estimated by an optical-sensing image. Section 2 reviews the development of deep learning and semantic segmentation. Section 3 is the Deeplab principle. Section 4 describes the specific method used in oil spills and the end-to-end network. Section 5 outlines the experiment that verified the feasibility of our method in oil-spill monitoring.

## 2. Related Work

Nowadays, deep learning is widely used in the field of computer vision. There are several general architectures for deep learning, such as Visual Geometry Group (VGG) [14] and Resnet [15]. VGG was proposed by the Computer Visual Group of the University of Oxford. It explored the relationship between the depth of a convolutional neural network and its performance. A deep neural network was successfully constructed by repeatedly stacking small convolutional layers and max-pooling layers. The advantage was that, although the network was deepened, it did not bring about the parameter-explosion problem, and its learning ability was strong. However, it took up more memory due to the increase in the number of layers and parameters. Resnet proposed a residual module and introduced an identity map to solve the degradation problem in the depth grid. Compared with VGG, it could make the grid as deep as possible. Resnet also reduced the error rate and had low computational complexity; we based our model on Resnet.

A semantic-segmentation method based on deep learning rapidly developed. There were three main methods. The first approach was based on upsampling segmentation. A convolutional neural network (CNN) lost some details when sampling. This process was irreversible, which resulted in low image resolution. The advantages of upsampling methods could compensate to a certain extent for some missing information and give a more accurate segmentation boundary. For example, Long J. et al. proposed a fully convolutional network (FCN) [16] that was applied to semantic segmentation and achieved high accuracy. However, it was insensitive to image details, not considering the relationship between pixels and lacking spatial consistency. The second segmentation approach was based on a probability-graph model, such as the second-order CRF, employed to smooth noisy segmentation maps. The model coupled neighboring nodes that favored same-label assignments to spatially proximal pixels. In addition, at this stage of DCNN, the score map was usually very smooth. The goal was to restore a detailed local structure. In this situation, a traditional approach missed fine structures. To overcome these limitations, a fully connected CRF could capture fine details. The third segment approach was based on improved feature resolution, recovering reduced resolution in deep convolutional neural networks to obtain more contextual information, such as in Deeplab. We combined DCNN with probability-map models and adjusted the resolution by atrous convolution to expand the receptive field. Multiscale feature extraction was performed by the atrous spatial pyramid pooling (ASPP) model to obtain global and local features. Then, we optimized the edge with the fully connected CRF to improve the results. Compared with the first two methods, the segmentation effect was improved. However, Deeplab first used the DCNN for rough segmentation, and then the fully connected CRF to perform fine segmentation, which could not achieve an end-to-end model.

The contribution of this paper was to realize an end-to-end connection between the DCNN and the fully connected CRF, considering the fully connected CRF as an RNN. We improved the accuracy and speed of the model.

## 3. Deeplab

**Figure 1:**
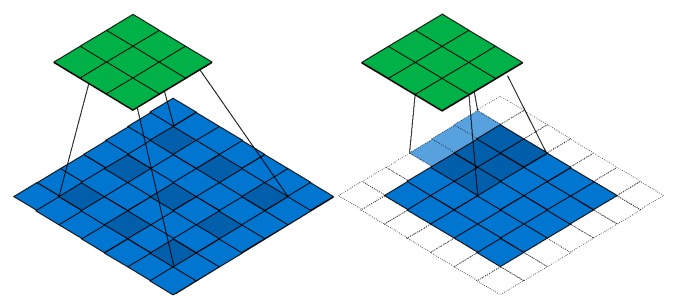
Principle of atrous convolution.

We used the method of atrous convolution [10] that extends the standard network-convolution operation. By adjusting the receptive field of the convolution filter, multiscale context information was captured. Then, it outputted features with different resolutions. Considering one-dimensional signals, output *y* of atrous convolution of a 1D input signal was defined as Equation (1); *w*[*k*] is the filter of length *K*. Figure 1 is the principle of a 2D convolution structure.

(1) $$y[i]=\sum_{k=1}^{K}x[i+r\cdot k]w[k]$$

**Figure 2:**
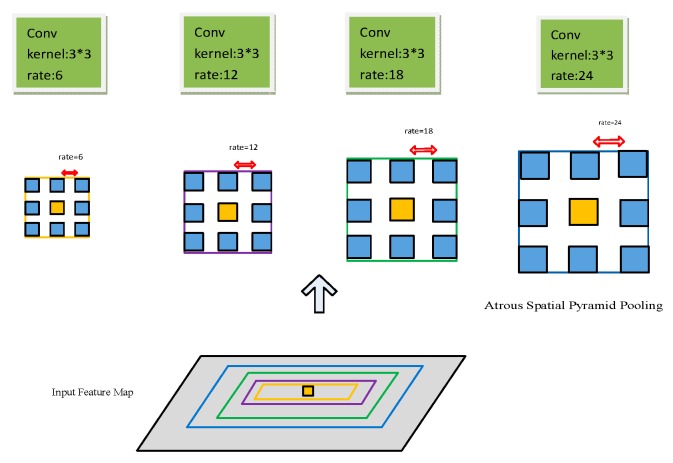
Atrous spatial pyramid pooling (ASPP) principle.

The model was based on Resnet. It transformed the fully connected layers of Resnet into convolutional layers. It used atrous convolution instead of the convolution kernel of the subsequent convolutional layer. Then, it needed to fine-tune the Resnet weight. In this way, Deeplab improved the resolution of the output feature map and enlarged the receptive field. The next step was multiscale extraction; ASPP is shown in Figure 2. In the given input characteristic graph, we used rate = (6, 12, 18, 24) 3 × 3 atrous-convolution parallel sampling. The results of each atrous-convolution branch sampled by ASPP were fused; then, we obtained the final prediction result. In fact, this scaled the image in different degrees through different types of atrous convolution. Deeplab achieved better segmentation results, but in ASPP, when the rate was larger, it was close to the size of the feature map. The 3 × 3 convolution degenerated into 1 × 1 convolution, so we changed the rate to (6, 12, 18). Then, we added a batch-normalization (BN) layer in ASPP, which could improve the generalization ability of the network and speed up network training to reduce time consumption.

## 4. Sea-Oil-Spill Monitoring

There are some common problems in monitoring an oil-spill area on the basis of multisource remote-sensing images, such as spot noise and uneven intensity. Many dark areas in SAR images are classified as oil-spill areas. Sea clutter in optical remote-sensing images affects target detection. On the basis of Deeplab, an input image was roughly segmented by DCNN. Then, we improved the fully connected CRF, which was output as an RNN. This step was to finely segment the image. We realized an end-to-end connection between DCNN and fully connected CRF, combining the DCNN with the improved fully connected CRF model in a unified end-to-end framework. Then, we improved the atrous-convolution rate in the ASPP and added a BN layer to increase grid-training speed. Finally, the fully connected conditional random field algorithm based on the average-field-approximation theorem was improved, obtaining an end-to-end connection with the DCNN.

### 4.1. Fully Connected Conditional Random Fields

We introduced the fully connected CRF in this section. Pixel labels were modeled as random variables to form Markov random fields under global observation. We set the picture to *I*; *x_i_* is the label of pixel *i*, taking the value from *L_i_*, random variables *x_1_, x_2_, …, x_N_* formed vector *X*; and *N* is the image-pixel number. *I* and *X* could be modeled as CRF, as shown in Equation (2).

(2) $$P(X=x|I)=\frac{1}{Z(I)}exp(-E(x|I))$$

Gibbs distribution is shown in Equation (3).

(3) $$E(x)=\sum_{i}\psi_{u}(x_{i})+\sum_{i<j}\psi_{p}(x_{i},x_{j})$$

where $\sum_{i<j}\psi_{p}(x_{i},x_{j})$ are the pairwise potentials, measuring the cost of assigning labels *x_i_*, *x_j_* to pixels *i*, *j*; and $\sum_{i}\psi_{u}(x_{i})$ are the unary energy components, measuring the cost of the pixel *i* taking the label *x_i_*. This paper was obtained by DCNN.

In the pairwise energies, as shown in Equation (4), similar pixels were more likely to label the same label. *f_i_* is the feature vector of pixel *i*; $w^{\left(m\right)}$ is the linear combination of weights; function $\mu (x_{i},x_{j})$ acts as punishment, and it calls the label compatibility function. Each $k_{G}^{m}$ is a Gaussian kernel applied to the feature vectors.

(4) $$\psi_{p}(x_{i},x_{j})=\mu (x_{i},x_{j})\sum_{m=1}^{M}w^{\left(m\right)}k_{G}^{\left(m\right)}(f_{i},f_{j})$$

### 4.2. Conditional Random Field (CRF) as Recurrent Neural Network (RNN)

According to the above formula, the result of labeling could be obtained by minimizing *E(x)*, but the process is complicated. The approximate distribution of the fully connected CRF is used to calculate maximal posterior edge inference [17]. *Q(X)* is the approximate distribution of *P(X)*. The average field-approximation reasoning iterative algorithm steps are shown in Algorithm 1.
**Algorithm 1.** Average-field algorithm. Mean-field in dense CRFs, broken down to common DCNN operations. Note: CRF, conditional random field; DCNN, deep convolutional neural network.$Q_{i}\left(l\right)\leftarrow \frac{1}{Z_{i}}\exp\left(U_{i}\left(l\right)\right)\mathrm{for}\;\mathrm{all}\;i$                   Initialization**While not converged, do**${\tilde{Q}}^{\left(m\right)}\left(l\right)\leftarrow \sum_{j\neq i}k^{\left(m\right)}\left(f_{i},f_{j}\right)Q_{j}\left(l\right)\mathrm{for}\;\mathrm{all}\;m$             Message${\overset{\vee }{Q}}_{i}\left(l\right)\leftarrow {\sum_{m}w^{\left(m\right)}\tilde{Q}}_{i}^{\left(m\right)}\left(l\right)$         Weighting Filter Outputs${\overset{\wedge }{Q}}_{i}\left(l\right)\leftarrow \sum_{l^{\prime }\in L}\mu \left(l,l\prime \right){\overset{\vee }{Q}}_{i}\left(l\right)$    Compatibility Transform${\overset{\vee }{Q}}_{i}\left(l\right)\leftarrow U_{i}\left(l\right)-{\overset{\wedge }{Q}}_{i}\left(l\right)$       Adding Unary Potentials$Q_{i}\leftarrow \frac{1}{Z_{i}}\exp\left({\overset{\vee }{Q}}_{i}\left(l\right)\right)$         Normalizing**end while**

The first step is an initialization, which is equivalent to applying a softmax function on the *U* of all the labels on each pixel; it does not need any parameters. This step can be seen as the softmax layer of neural networks. The second step is message passing, which is achieved by applying *M* Gaussian filters to *Q* values. Gaussian filter coefficients are derived on the basis of pixel locations and Red, Green, Blue (RGB) values. This can be regarded as the convolutional operation. The next step is weighting the filter outputs. For each class label, the weighted sum of the previous Gaussian filter output is computed. This process can be regarded as usual convolution with a 1 × 1 filter. The fourth step is compatibility transformation. If assigned a different label to a pixel with similar properties, it is penalized. This can be viewed as another convolution layer, and the spatial receptive field of the filter is 1 × 1. The fifth step is adding unary potentials; the output of the compatibility-transformation stage is subtracted elementwise from unary inputs *U*. Result *U* and global probability transfer result ${\overset{\wedge }{Q}}_{i}\left(l\right)$ determine the final probability. The last step is normalization. Results of the fifth step are normalized to the initial probability of the next RNN iteration. This can be regarded as another softmax operation, and it has no parameters.

In this paper, we improved the second and third steps. The original Gaussian kernel considered the location vector and color vector of *x*, *y*, that is, the Gaussian kernel was 2. In fact, the color vector determined the prior probability of the classification in the DCNN layer, so the Gaussian distance of the color vector could be ignored, and it only considered the location difference; the Gaussian kernel was 1. The farther the distance was, the smaller the difference was. We proposed an improved method by combining the full map distance weight and the network-training method. The second and the third steps combined the probability transfer and weight adjustment into a new algorithm, which was equivalent to the convolution operation. In Equation (5), *a_i_* is the distance weight, *l* is the class, and *Q_j_*(*l*) is the class probability for each point.

(5) $${\bar{Q}}_{i}(l)=\sum_{i\neq j}a_{i,j}Q_{j}(l)$$

**Figure 3:**
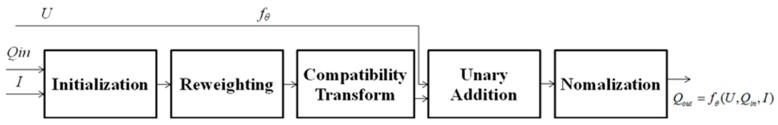
Reasoning algorithm iterative process.

The process of one iteration is shown in Figure 3, which could be expressed as multiple convolutional-neural-network layers. We used function $f_{\theta }$ to denote the transformation done by one mean-field iteration. Multilayer average field iteration could repeat the above implementation process, and each iteration came from the results of previous iterations. This process was equivalent to an RNN. The network was given by Equations (6–8). The initial value of *H_1_(t)* was the result of DCNN normalization, and *H_2_(t)* was the one iteration process of CRF. *H_1_(t),* except the initial value, was the output of the last *H_2_(t)*; *Y(t)* was the output of *T* times iteration; and *T* was mean-field iteration numbers. When the specified number of iterations *T* was not reached, the iteration was continued. If *t* = *T*, output *H_2_(t)* was the final iteration result.

(6) $$H_{1}\left(t\right)=\{\begin{array}{l} \mathrm{softmax}\left(U\right),\;t=0\\ H_{2}\left(t-1\right),\;0<t\leq T \end{array}$$

(7) $$H_{2}\left(t\right)=\{f_{\theta }\left(U,H_{1}\left(t\right),I\right),\;0\leq t\leq T$$

(8) $$Y\left(t\right)=\{\begin{array}{c} 0,\;0\leq t\leq T\\ H_{2}\left(t\right),\;t=T\; \end{array}$$

**Figure 4:**
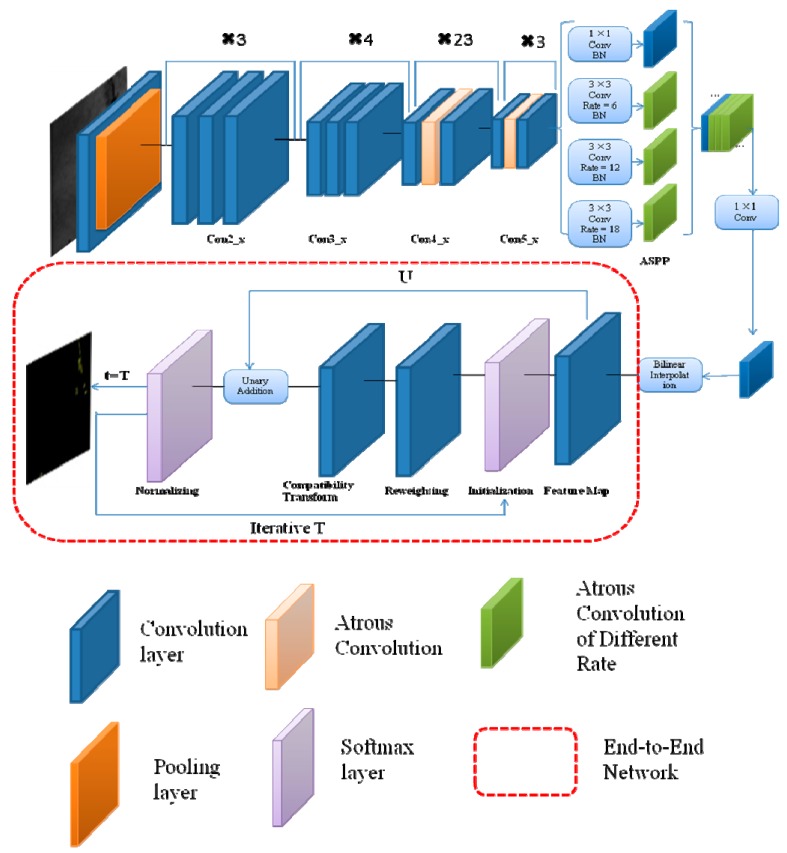
The end-to-end algorithm flow chart.

**Figure 5:**
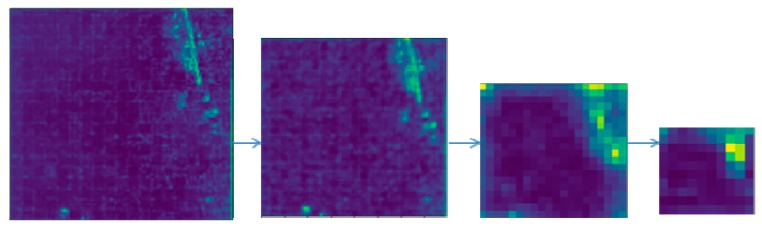
Multiscale ASPP visualization results.

Through the above improvements, the end-to-end algorithm structure is shown in Figure 4. First, the input image was processed by the Resnet network. It changed the middle layer of Con3_x and Con4_x to atrous convolution. Second, feature maps were obtained by different atrous-convolution rates in ASPP. The BN layer increased the training speed and the generalization ability of the network. The convolutional-neural-network visualization of ASPP is shown in Figure 5. When the receptive field was small, image details were extracted. Otherwise, it extracted the abstract features of the image. Then, it outputted the feature map by bilinear interpolation, which provided CRF unary potential. It was connected with the recurrent neural network. Finally, after entering the RNN, it needed to be iterated *t* to leave the loop. End-to-end training was performed using backpropagation and stochastic-gradient-descent algorithms. Once it exited the loop, the softmax layer terminated the network. Then, the network outputted the classification results. This algorithm unified the advantages of DCNN and fully connected CRF and achieved end-to-end connection.

## 5. Experiment

In the experiments, we detected an oil-spill area on the sea surface on the basis of a multisource remote-sensing image. We compared our method with other state-of-the-art methods. In this way, we verified the superiority of the model. The experiment computer was configured with an Intel i7 processor, NVDIA RTX2080Ti (Beijing, China), and 16 GB memory. We established a high-quality SAR image and optical remote-sensing-image dataset. The remote-sensing image came from the high-resolution Quickbird, the Worldview remote-sensing satellite, and Google Earth. The SAR image came from a C-band radar SAT-2 polar meter. There were 4200 oil-spill images. The dataset could be divided into three types: background, oil-spill area, and ship. Then, we randomly selected 60% of the images as the training set, 20% as the verification set, and 20% as the test set. Because training needs many pictures, we enhanced the data. It rotated each image by 90°, 180°, and 270°. Then, we obtained a dataset containing 8400 images. In the experiment, we used a poly strategy in the training process, as shown in Equation (9). The number of iterations in the deep convolutional neural network was set to 20,000, and the batch size was 20. “Epoch” means that all samples in the training set were trained once. There were 80 iterations in this experiment. The initial value of the learning rate was 0.001. When the learning rate was too large, it became unstable when converging to the optimal position. So, the learning rate should exponentially decrease with the training process. We used a weight decay of 0.0005 and a momentum of 0.9. Experiments included the classification results of the oil spill, mean intersection over union (mIOU) analysis, time analysis, and the calculation of the oil-spill area.

(9) $$poly={\left(1-\frac{iter}{max\_iter}\right)}^{power}$$

where power is the parameter, and the value is 0.9, iter represents the number of iterations, and max_iter represents the maximal number of iterations.

### 5.1. Oil-Spill Classification Result

This experiment compared the sea-surface oil-spill segmentation of an optical remote-sensing image and a SAR image. As shown in the figure below, the comparison methods were Deeplab, Semantic Projection Network (SPNet), and the method proposed in this paper.

**Figure 6:**
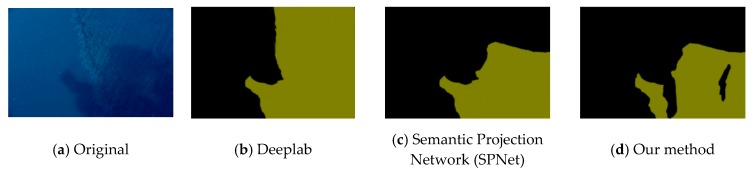
Optical remote-sensing-image of oil-spill segmentation results.

**Figure 7:**
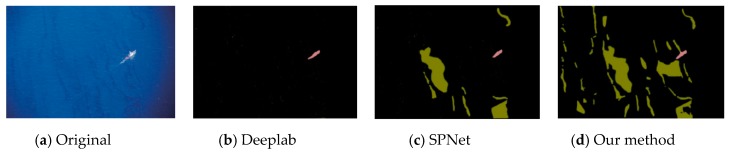
Optical remote-sensing-image of oil-spill segmentation results.

**Figure 8:**
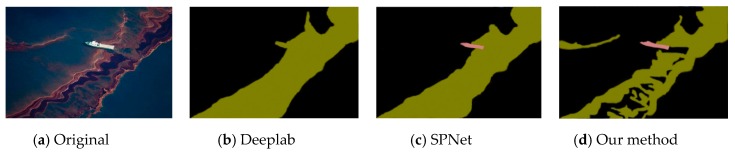
Optical remote-sensing-image of oil-spill segmentation results.

Figure 6, Figure 7 and Figure 8 are the segmentation results of the oil spill on the sea surface of the optical remote-sensing image. There contained no dark areas, such as the wind-shadow area, upwelling zones, or sea clutter ship wake. So, it could effectively eliminate the interference of dark areas. An oil spill has a different performance with seawater. Figure 6a is the original picture, where the oil-spill area is more obvious, but sea clutter interfered. Figure 6b shows the Deeplab result, which classified some sea-clutter errors into oil-spill areas. SPNet (Figure 6c) correctly classified part of the oil-spill area, but accuracy was poor. The results of our method, shown in Figure 6d, could accurately classify sea-clutter and oil-spill areas, capturing the fine details of the oil spill. The oil-spill area in Figure 7a was more dispersed and not obvious. Deeplab only correctly classified the ship. The proposed method in this paper is shown in Figure 7d. Compared with Figure 7b,c, our method’s classification was the best because our method realized an end-to-end connection, improving segmentation accuracy. As shown in Figure 8, Deeplab classified the ship as part of the oil spill. Both SPNet and our method correctly classified the ship and the oil-spill area. However, our method could strongly segment the target, and it achieved good results.

**Figure 9:**
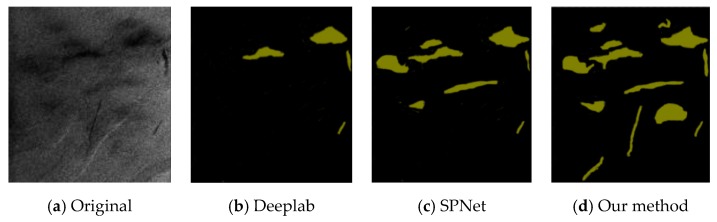
Synthetic-aperture-radar (SAR) image of oil-spill segmentation results.

**Figure 10:**
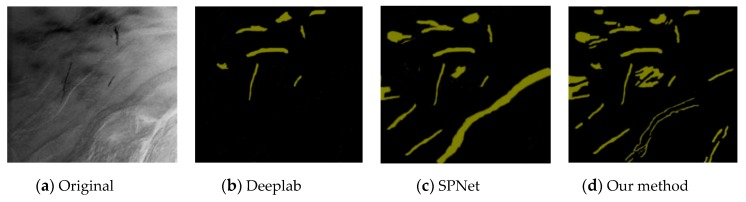
SAR image of oil spill segmentation results.

**Figure 11:**
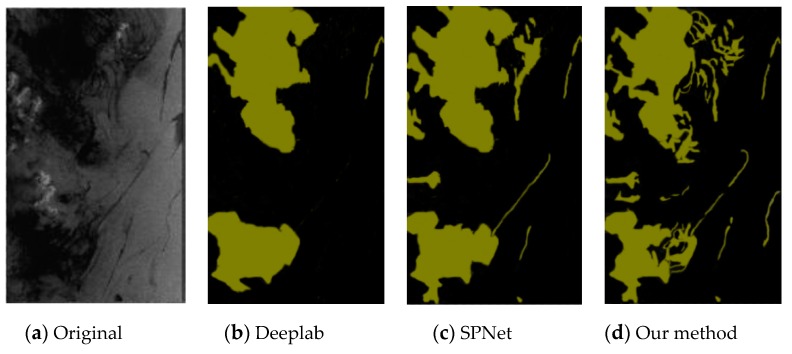
SAR image of oil-spill segmentation results.

Figure 9, Figure 10 and Figure 11 are the results of oil-spill classification for SAR images, which have inherent speckle noise. The characteristics of different regions were also not uniform, which led to classification difficulty. As could be seen from the three figures, Deeplab could only correctly classify the serious oil-spill area, that is, the darker part of the image. Although SPNet correctly classified some oil-spill areas, it was blurry at the oil-spill edge. Our segmentation results were better than those of the first two models. However, it was still not very good to identify similar oil-spill areas in the SAR images by using the deep-learning method. We aimed to improve the database in the future.

Through experiment analysis, the method proposed in this paper was found to be suitable for both optical remote-sensing and SAR images; the segmentation effect was also good.

### 5.2. Mean Intersection over Union Analysis

In this section, we compared our method with other state-of-the-art methods on the established dataset. The evaluation index was mean intersection over union (mIOU), which is the standard measure of semantic segmentation, as shown in Equation (10). The comparison results are shown in Table 1. The lowest mIOU value was FCN-8s. The detection effect was poor because it was not a deep network structure. The Deeplab value was 76.5. The value of SPNet was after our model, reaching 78.9. Compared with other state-of-the-art methods, the results of our method were better. The mIOU was 82.1.

(10) $$mIOU=\frac{1}{k+1}\sum_{i=0}^{k}\frac{p_{ii}}{\sum_{j=0}^{k}p_{ij}+\sum_{j=0}^{k}p_{ji}-p_{ii}}$$

where *k* is the category, *i* is the true value, *j* is the predicted value, *p_ij_* means to predict class *i* as class *j*.

In this experiment, we compared whether there existed a BN layer, ASPP, and atrous convolution on the dataset; results are shown in Table 2. Adding a BN layer had little effect on segmentation-result accuracy because a BN layer is mainly used to speed up network training and reduce time consumption. When atrous convolution was used instead of traditional convolution operation, the result was significantly improved because an atrous-convolution operation expanded the receptive field and improved output feature-map resolution. The mIOU of the method without using CRF was 80.8. The CRF could refine the edge of the picture and increase accuracy. By using the improved mean-field theorem and the end-to-end connection method, segmentation accuracy was improved. To sum up, the method proposed in this paper significantly improved segmentation results.

We compared the effect of CRF iterations on the experiment results, as shown in Table 3. When the number of iterations was 5 or more, the mIOU was not significantly improved. We considered that multiple iterations took a lot of time. We used *T* = 5 in this paper.

**Figure 12:**
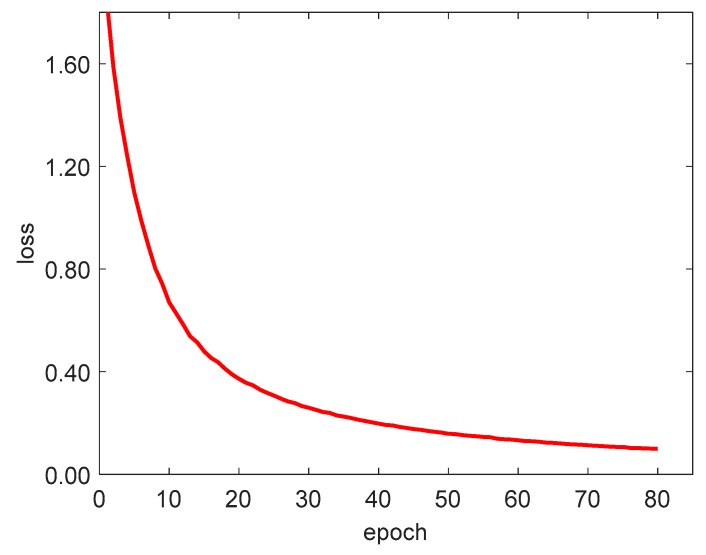
Loss function curve.

We used the cross-entropy cost function to calculate the loss. Its definition is shown in Equation (11). Cross entropy represents the difference between true and predicted probability distribution. In deep learning, true distribution was determined. The smaller the cross entropy was, the better the prediction effect was. The loss-function curve is shown in Figure 12.

(11) $$H(p,q)=-\sum_{i=1}^{n}p(x_{i})\log(q(x_{i}))$$

where *p*(*x_i_*) represents true probability distribution, and *q*(*x_i_*) represents predicted probability distribution.

### 5.3. Runtime Analysis

We compared the runtime of the established dataset with other state-of-the-art methods, as shown in Table 4. It could be seen from the table that the Deeplab took up to 1.4 s. The reason was that there was no end-to-end connection in the model. The shortest time was FCN-8s. The model proposed in this paper took a relatively short time. Our method and FCN-8s were in the same order of magnitude, and the detection accuracy was guaranteed.

### 5.4. Oil-Spill-Area Calculation

The calculation of the oil-spill area on the sea surface could be the degree of sea-surface pollution. It is also the basis for future sea pollution, which is of great significance. In this experiment, the oil-spill area was estimated by satellite resolution and the number of pixels in the oil-spill area. Because of the high resolution of the optical remote-sensing image, it usually refers to a ground area of about 1 × 1 m, represented by one pixel. The oil-spill area could be obtained by multiplying the number of pixels in the oil-spill area by the square of the satellite resolution, as shown in Equation (12).

(12) $$S_{\mathrm{os}}\approx N_{os}\times R^{2}$$

where *S_os_* is the area of the oil spill, *N_os_* is the number of pixels on the oil spill, and *R* is the satellite resolution.

The oil-spill area of the optical remote-sensing image is shown in Table 5.

## 6. Conclusions

In this paper, an oil-spill area was monitored by SAR and optical images. The influence of a complex ocean environment on multisensor images, such as dark areas in SAR images, could be eliminated. This paper was based on the DCNN and a fully connected CRF. Instead of the max-pooling layer, we used atrous convolution and added a BN layer to the multiscale convolution layer. It realized an end-to-end connection with the fully connected CRF. We obtained a deep network with the characteristics of both DCNN and the fully connected CRF. This overcame the problem of the poor classification of satellite images for oil-spill monitoring and improved the ability to capture fine target details. The mIOU of the established dataset in this paper reached 82.1, and the result of remote-sensing image classification was good. In addition, the oil-spill area was effectively estimated by optical imaging. This was of great significance to the restoration of a sea environment and sea-pollution inspection. In the future, we intend to integrate the limitations of the methods proposed in this paper and improve running time while continuing to improve classification accuracy in a relatively short time.

## Figures and Tables

**Table 1:** Comparison of different models mIOU.

Method	Mean Intersection Over Union (mIOU)
FCN-8s [18]	60.1
DeepLab-MSc [10]	70.3
CRF-RNN [17]	71.8
Deeplab [10]	76.5
HDC [19]	74.8
SPNet [20]	78.9
H-ReNet+DenseCRF [21]	76.8
Oxford HO CRF [22]	77.9
Proposed	82.1

Note: FCN, fully convolutional network; DeepLab-MSC, Deeplab multiscale; CRF-RNN, conditional random field-recurrent neural network; HDC, hybrid dilated convolution; SPNet, semantic projection network; Oxford HO CRF, Oxford Higher Order conditional random field.

**Table 2:** Employing model on a dataset. batch-normalization (BN).

BN	Atrous Spatial Pyramid Pooling (ASPP)	End-to-End Connection without CRF	Atrous Convolution	CRF	End-to-End Connection with Improved CRF	mIOU
✓	✓					74.9
✓	✓				✓	79.2
✓	✓	✓	✓			80.8
	✓		✓		✓	81.9
✓	✓		✓	✓		81.7
✓	✓		✓		✓	82.4

**Table 3:** Effect of CRF iterations.

Iteration	1	2	3	4	5	6	7	8	9	10
mIOU	79.5	80.0	81.2	81.7	82.1	82.2	82.3	82.4	82.5	82.6

**Table 4:** Time-consuming analysis of different models.

Method	Runtime
FCN-8s	0.55 s
Deeplab	1.4 s
SPNet	1.25 s
DeepLab-MSc	1.2 s
Proposed	0.8 s

**Table 5:** Oil-spill area of the visible remote-sensing image.

Satellite	Pixels	Satellite Resolution	Oil-Spill Area
Optical Image 1 (Quickbird)	78,112	0.61 m	29,065.5 m^2^
Optical Image 2 (Worldview)	29,774	0.5 m	7443.5 m^2^
Optical Image 3 (Worldview)	61,548	0.5 m	15,387 m^2^

## Data Availability

The data used to support the findings of this study are available from the corresponding author upon request.
